# Practical Notes on Cutaneous Subjects

**Published:** 1874-04-15

**Authors:** Tilbury Fox


### PRACTICAL NOTES ON CUTANEOUS SUBJECTS.

By Tilbury Fox, M.D., F.R.C.P.

*From the London Lancet, Mar.*, 1874.

The Diagnostic Sign of Phthiriasis.—Considering the great practical value of the pathognomonic lesion of phthiriasis (or the disease due to lice), which I described some two or three years since, I have been more than surprised that those who profess to study dermatology in England should have not thought it worth while to have made themselves acquainted with it. I have had the pleasure, from time to time, of demonstrating this lesion to a number of foreign dermatologists who have visited my clinic at University College Hospital, and they have fully admitted the significance of the sign in question. There are many cases in which it is impossible to detect pediculi, where they are really present; and in these cases the lesion to which I refer will be detected very easily, and is the sure evidence of the attack of pediculi upon the skin.

It is easy to mistake the characteristic lesion; and in such cases the observer will, of course, affirm that the lesion I describe is not reliable. The lesion which I say is characteristic, is not a bite or a scratch; it is the opening of a follicle dilated by the proboscis of the pediculus, and showing in its center a speck of at first bright-red blood, which soon acquires a darker hue.

This hæmorrhagic speck, or "lesion," is not raised to the feel or the eye. It looks like a circular, cup-shaped depression, about the size of the blunt point of an ordinary pin, with a well-marked circumferential edge (a dilated follicle), and a black dot in the center. It may be confounded with scratched hyperæmic follicles, or papillæ, or minute excoriations. The former are raised, and on being examined with the magnifying glass, are seen not to be round, but to have ragged edges, and to present a bleeding surface; the excoriations are irregular in shape, and want the look of the dilated follicle-mouth, with the speck of blood in the center.

The fact is, the pediculus has no mouth; it does not bite. It has a proboscis which it pushes into a follicle to reach a capillary vessel. In the act of sucking blood away, the mouth of the follicle is dilated, and when the proboscis is withdrawn, the blood wells up to fill the dilated orifice.

I consider it altogether unnecessary to search for pediculi amongst the clothes of the patient.

There are many cases of phthiriasis in the young, where pediculi are with great difficulty detected, from whatever cause this may be, and in which the recognition of the lesion I now refer to sets all doubt at rest, and, by leading to a correct diagnosis, secures a speedy cure to the patient.

THE TREATMENT OF NON-PARASITIC SYCOSIS.—No disease, I take it, is more unsatisfactory to treat than the common inflammation of the hair-follicles of the beard and whiskers, to which the term sycosis non-parisitica is applied. On the continent, especially in Germany, the practitioner is advised to adopt epilation, and to apply some simple astringent ointment; and there is a great disposition now-a-days to regard epilation as *the* remedy for the disease under notice. The reason for epilating is variously stated. Some affirm that the inflammation in sycosis is caused by a premature development of a new hair in the follicle, and that it is necessary in its cure to rid the follicle of the old hair. Others think that suppuration extends to the root of the hair, and that epilation relieves the tension of the parts and permits the exit of the pus. The first explanation will not bear examination. The second is true, in part. In non-parasitic sycosis inflammation travels downwards, and may reach the bottom of the follicle, the root of the hair being bathed in pus, whilst the hair is loosened from its surrounding connections, and lies, as it were, a dead piece of tissue in the follicle. In such cases, epilation does but get rid of the loosened hair; and its extraction allows the escape of pus that would otherwise be pent up. But in many cases the inflammation does not proceed to the extent of causing suppuration in the deep part of the follicle; the hairs are not loosened in the follicles; and their extraction gives great pain, and can do no good. Epilation is, therefore, a fit procedure only at a certain stage of sycosis—if the skin is much inflamed, the follicles freely suppurating, and the hairs are thereby loosening or loosened in them.

The treatment which I have found most successful may be summed up as follows: In the early stage, when the follicles are very hyperæmic, saline aperients, in persons of full habit; or aperient tonics, such as sulphate of magnesia with sulphate of iron, in those who are debilitated; together with hot fomentations, and simple, soothing applications which exclude the air, locally. When there is free suppuration, the same internal remedies, together with the removal, by epilation, of the loosened hairs from freely-suppurating follicles, and the application of mild astringents, such as zinc lotions and ointment; and, lastly, in the sub-acute or chronic stage, where there is only a suppurating follicle here and there, but mostly a number of indurated tubercles—i.e., follicles thickened by hyperplasic growth of the connective tissue—a course of Donovan's solution, together with, locally, hot fomentation, and the application of a weak nitrate-of-mercury ointment (a drachm and a half to an ounce) night and morning. Of course, for persons of scrofulous constitutions, cod-liver oil and iron are to be given, in combination with alterative remedies. I fully admit that the exhibition of Donovan's solution is, in great part, an empirical proceeding; but I prefer it to any other remedy, and have reason to speak with confidence as to its efficacy in sycosis, when employed in the way, and at the particular stage, above indicated. Lastly, I may add that it is an easy matter to do harm in sycosis, by the injudicious use of local stimulants, which intensify the hyperæmia and the hyperplasic thickening; and I believe this to be the radical fault in the treatment of sycosis.

---

MORE than a hundred people are drinking warm blood at the Brighton, Mass., abattoir, for various diseases, and there is talk of building a hotel to accommodate the patients.

